# Internet tools to enhance breast cancer care

**DOI:** 10.1038/npjbcancer.2016.11

**Published:** 2016-04-27

**Authors:** Shlomit Strulov Shachar, Hyman B Muss

**Affiliations:** 1 Lineberger Comprehensive Cancer Center, Department of Medicine, University of North Carolina, Chapel Hill, NC, USA; 2 Division of Oncology, Rambam Health Care Campus, Haifa, Israel

## Abstract

Internet tools have become a great aid in the daily practice of physicians who treat breast cancer patients. In cancer care there are frequent and important intersections where major decisions need to be made; these include (1) whether or not to give chemotherapy; (2) how much toxicity to expect, and (3) the life expectancy of the patient, considering non-breast cancer comorbidities. These decisions can be made more accurately using calculators based on data sets of thousands of patients as opposed to physician intuition. Such tools also help patients and caregivers in optimal decision making, as they estimate the absolute benefits and risks of treatment. In this perspective we describe selected internet sites that are useful across several domains of care, including the potential benefits of different adjuvant regimens for early breast cancer, prognosis after neoadjuvant therapy, prognosis for ductal carcinoma *in situ*, and toxicity and life expectancy estimates. We review the variables required to use the tools, the results obtained, the methods of validation, and the advantages and disadvantages of each tool.

## Introduction

In the past decade there has been increased usage of online tools to determine the value of adjuvant systemic therapies for breast cancer—including neoadjuvant therapy—to estimate life expectancy, to predict outcomes for patients with ductal carcinoma *in situ* (DCIS) based on the varied treatment options, and to estimate chemotherapy-related toxicity in older patients. Having an accessible online tool that can estimate and personalize the benefits of different treatment outcomes for individual patients has become a great help in daily practice. There are currently many resources available; this perspective will focus on several which we feel are most helpful. In addition to describing the strengths and weaknesses of each tool’s application, we will discuss how they were validated. A detailed list of our preferred sites is shown in Table 1.

### Tools for systemic adjuvant therapy

#### Adjuvant!

Adjuvant! (https://www.adjuvantonline.com/) is a groundbreaking program that is probably the most widely used tool for estimating the benefits of adjuvant endocrine therapy and chemotherapy.^1^ The tool assesses individual patient risk of recurrence and death at 10 years. Mortality risk is based on surveillance, epidemiology, and end-results (SEER) data for women aged 36–69 years, and estimates of the efficacy of adjuvant therapy from data from the Early Breast Cancer Trialists' Collaborative Group. Entering information on age and selected tumor characteristics (tumor size and grade, number of positive axillary nodes, and hormone receptors status) allows for prediction of the 10-year risk of relapse-free and overall survival. A strength of this tool is that one can add a rough estimate of the effect of comorbidity on survival to the model. This allows the clinician to determine the benefits of treatment when patients have major competing causes of mortality—in addition to their breast cancer mortality risk. Adjuvant!’s strength also lies in the fact that it provides details on deaths from both breast cancer and non-breast cancer causes. This latter information is especially important in older patients, for whom 10-year mortality is frequently dominated by non-breast cancer related events.

Despite these strengths, Adjuvant! has several limitations. The relapse estimates include local–regional recurrence as well as distant metastases; this is important as the proportions of both may vary greatly depending on stage and tumor phenotype. In addition, data from SEER were not available for HER-2 status, and the benefits of adjuvant trastuzumab are not available in this model. Validation also poses a problem, as some studies of the model have not been consistent. Although a Dutch study confirmed the accuracy of the tool in the European population (*N*=5380),^2^ a British study (*N*=1065) found that in a high percentage of patients survival was overestimated.^2,3^ Another validation in an elderly population (*N*=2012) showed there was an overestimation of the added value of chemotherapy for older patients and those younger than 40 years.^2,4^

#### PREDICT

The PREDICT tool (http://www.predict.nhs.uk/) was developed using cancer registry data from 5,694 patients in the UK.^5^ Validation of the model was made on 5,000 other patients from the U.K. and 3140 patients from Canada.^6^ An estimation of therapy and prognosis of HER 2 tumors was later incorporated.^7^ The PREDICT tool utilizes data on patient age and tumor characteristics (the mode of detection (i.e., screening versus discovery of a palpable mass), size, grade, ER status, and KI67 status) to provide a choice for estimating the value of endocrine therapy alone, or endocrine therapy and second-generation chemotherapy (anthracycline-containing, >4 cycles or equivalent) versus third-generation (taxane-containing chemotherapy regimens).^8^ The predict model allows one to estimate the effects of adjuvant endocrine and chemotherapy treatment on survival at 5 and 10 years, but there is no estimate of relapse and it does not account for non-breast cancer causes of mortality in the overall survival estimate. However, unlike Adjuvant!, the PREDICT model can estimate the benefits of anti-HER2 therapy in patients with HER-2 positive tumors.^7^ In addition, a recent study has validated this tool’s ability to provide accurate estimates of the potential benefits of treatment at 5 years for older patients.^9^ Table 2 provides several scenarios showing the effects of treatment selection on survival using the PREDICT model.

#### CancerMath

CancerMath (http://www.lifemath.net/cancer/breastcancer/therapy/) utilizes tools that estimate the probability of having positive lymph nodes (based on age and tumor characteristics), breast cancer mortality, and the potential benefits of treatment with endocrine therapy and chemotherapy. Estimates are based on SEER data (*N*=362,491) and include HER-2 status, tumor size, nodal involvement, tumor phenotype, and grade.^10^

#### Oncotype DX

The Oncotype DX website (https://online.genomichealth.com/Login.aspx) provides two diagnostic tools that analyze recurrence scores and take into account the type of endocrine therapy (tamoxifen or an aromatase inhibitor) as well as patient age, tumor size, and tumor grade, to further refine the estimates of endocrine therapy on 10-year metastases-free survival. This can result in small but potentially important changes in our understanding of metastatic relapse risk, and could help physicians make the decision of whether to offer chemotherapy. This combined score has resulted in classifying fewer patients as intermediate risk (17.8% vs 26.7%, *P*<0.001) and more patients as lower risk (63.8% vs 54.2%, *P*<0.001).^11^

#### Neoadjuvant chemotherapy outcomes tool

The neoadjuvant chemotherapy outcomes tool (http://www3.mdanderson.org/app/medcalc/index.cfm?pagename=bcnt) provides estimates of 5-year distant metastases-free and disease-specific survival after neoadjuvant treatment, and incorporates initial clinical stage before treatment, post-neoadjuvant pathological stage, estrogen receptor status, and nuclear grade.^12^

### Tools for treatment outcomes for patients with ductal carcinoma *in situ*

An online tool, the ‘Breast Cancer Nomogram: Ductal Carcinoma *In Situ* (DCIS) Recurrence’ has been developed at the Memorial Sloan Kettering Cancer Center (http://nomograms.mskcc.org/breast/DuctalCarcinomaInSituRecurrencePage.aspx) to predict in-breast recurrence risk after breast-conserving surgery.^13^ The program, takes into account many patient and tumor characteristics, including age, family history, presentation, tumor grade, presence of necrosis, surgical margins, year of surgery, and number of excisions, as well as the potential risk-reducing benefits of adjuvant breast irradiation and/or endocrine treatment. It provides both the 5- and 10-year probability of in-breast recurrence. This can be especially helpful to patients and physicians, since mortality with this diagnosis is extremely low and many patients may elect to forego radiation and endocrine treatment after reviewing the potential risks and benefits of each modality.

### Tools for predicting life expectancy

#### ePrognosis

The use of chronological age to predict life expectancy in older patients should be discouraged (http://eprognosis.ucsf.edu/calculators.php). There is great heterogeneity in the health status of older people that can result in dramatic differences in life expectancy in persons of the same chronological age. Comorbidity, nutritional status, physical and cognitive function, social support, and mental health status all are related to longevity. The average practicing oncologist has generally not been trained in geriatric assessment, and the ePrognosis website provides a series of tools for estimating life expectancy from non-breast cancer causes for older adults living in the community, a nursing home, or who are hospitalized.^14^ The average remaining life expectancy of women at different ages and with different levels of comorbidity is shown in Table 3 (patient 1 is older but healthier with better 10-year survival than patient 2 who is younger but sick). Clinical efforts to estimate life expectancy from non-breast cancer causes are valuable, since life expectancy is a key factor in making treatment decisions in older patients—especially decisions concerning chemotherapy. The site also has an extremely helpful palliative performance scale for outpatients with advanced cancer that takes into consideration ambulatory status, the patient’s level of daily activity and the need for self-assistance, oral intake, and level of consciousness.^15^ The tool has moderate discrimination but can be provide a reasonable estimate of an ill patient’s median survival in days.

### Toxicity-risk calculators for older patients

#### CARG

The Cancer and Aging Research Group (CARG) (http://www.mycarg.org) has developed a toxicity-risk calculator based on data from 500 patients with a variety of both early and late stage cancers. The calculator allows for prediction of grades 3–5 toxicities^16^ and the model includes standard clinical variables (gender, age, weight, height, serum creatinine, hemoglobin level, cancer type, chemotherapy treatment (dosage), and single agent or combination chemotherapy) as well as six variables attained via a short geriatric assessment (hearing status, number of falls, hearing problems, ability to take medications, ability to walk one block, and social activities limitations due to health or emotional problems). From these entries one can calculate a risk score that not only can reasonably predicts severe toxicity, but also is superior to performance status, a poor predictor.^17^

#### CRASH (Chemotherapy-risk assessment scale for high-age patients)

CRASH (http://moffitt.org/cancer-types--treatment/cancers-we-treat/senior-adult-oncology-program-tools) is a user-friendly tool to estimate the risk of severe chemotherapy toxicity based on the specific chemotherapy regimen, diastolic blood pressure, instrumental activities of daily living, lactate dehydrogenase, performance status, mini-mental status, and a mini-nutritional assessment. This tool was developed and validated on a cohort of cancer patients 70 years and older (*N*=512).^18^

### Conclusions

The tools we have discussed are readily available for use in daily practice and office staff can be trained to use these models and provide information to busy clinicians. Most of them have a user-friendly interface and can be used without registration and a password, which is a great advantage on a busy day. The tools used for assessing the benefits of adjuvant systemic therapy and the management of DCIS are frequently used in patients who have had tumor tissues sent for newer genetic-based assays. Such assays may provide more detailed information, especially in patients with node-negative, hormone receptor-positive, and HER-2-negative breast cancers,^19–23^ and in many patients the estimates from genetic-based assays are more appropriate for decision making.

The internet has given us the ability to rapidly use key clinical information at the point of patient contact to help make treatment decisions. The tools discussed in this review give physicians the opportunity to obtain relatively precise, up to date estimates of treatment effect, longevity, and toxicity that are likely to be more accurate than decisions made on intuition and experience alone. It is important, however, that those who have developed these tools and who will develop new tools in the future continually re-validate each tool as new data become available and make appropriate modifications as needed.

## Figures and Tables

**Table 1:** Web sites for breast cancer care

*Name*	*Details*	*URL/Link*
*Breast cancer predictive websites*		
Adjuvant! (adjvuvantonline.com)	Calculate benefits of adjuvant therapy for patients with breast cancer. Can add estimates of comorbidity to calculations.Registration and password needed	https://www.adjuvantonline.com/
CancerMath	Several tools for predicting survival at 15 years, estimating therapy benefit.	http://www.lifemath.net/cancer/breastcancer/therapy/
DCIS Recurrence Memorial Sloan Kettering	A tool for patients who had BCS for DCIS to predict the likelihood that their breast cancer will return in the same breast that was originally treated.	http://nomograms.mskcc.org/breast/DuctalCarcinomaInSituRecurrencePage.aspx
PREDICT	UK-derived tool which calculates benefits of adjuvant therapy for patients with breast cancer. Does not allow for comorbidity. Can calculate benefits for patients with HER-2-positive tumors.	http://www.predict.nhs.uk/
Oncotype DX^®^ tools	Tools to understand how hormonal therapy and pathological and clinical factors can be assessed with the Oncotype DX^®^ Breast Cancer Assay Recurrence Score result. Registration and password needed.	https://online.genomichealth.com/Login.aspx
Neoadjuvant Therapy Outcomes Tool MD Anderson Cancer Center	Calculates the anticipated 5-year distant metastasis-free survival and disease-specific survival for breast cancer patients following treatment with neoadjuvant chemotherapy. Pathological response also integrated.	http://www3.mdanderson.org/app/medcalc/index.cfm?pagename=bcnt
		
*Life expectancy prediction and geriatric oncology websites*		
ASCO University	A series of online modules that explore different care options for older patients, including those with breast cancer. Also has MOC course on geriatric oncology.	http://university.asco.org/geriatric-oncology
CARG (Cancer and Aging Research Group)	A group of researchers with major interest in geriatric oncology research. Opportunities for mentoring. Website includes online chemotherapy toxicity tool and geriatric assessment tools.	http://www.mycarg.org/
ePrognosis	A series of tools based on systematic review of literature that allows for estimation of life expectancy in older adults.	http://eprognosis.ucsf.edu/default.php
International Society of Geriatric Oncology (SIOG)	International organization that focuses on geriatric oncology. Website has useful links to geriatric oncology guidelines and other educational materials.	http://www.siog.org/
		
*Toxicity prediction websites*		
Moffitt Cancer Center Senior Adult Oncology Program Tools	Online tools for estimating chemotherapy toxicity (CRASH score) and other geriatric tools	http://moffitt.org/cancer-types--treatment/cancers-we-treat/senior-adult-oncology-program-tools
CARG (Cancer and Aging Research Group)	Online chemotherapy toxicity tool and geriatric assessment tools.	http://www.mycarg.org/

Abbreviations: ASCO, American Society of Clinical Oncology; BCS, breast-conserving surgery; CRASH, chemotherapy risk assessment scale for high-age patients; DCIS, ductal carcinoma *in situ*; HER2, human epidermal growth factor receptor 2; MOC, Maintenance of Certification; UK, United Kingdom.

**Table 2:** Survival benefit of adjuvant treatment in breast cancer by PREDICT

	*Patient 1*	*Patient 2*	*Patient 3*	*Patient 4*
*Patient and tumor characteristics*				
Age	42	55	72	38
Mode of detection	Symptomatic^a^	Screening	Symptomatic	Symptomatic
Tumor size (mm)	18	15	40	32
Tumor grade	3	2	2	3
Number of positive lymph nodes	3	0	2	1
Estrogen Receptor status	Negative	Positive	Positive	Negative
HER2 status	Positive	Negative	Negative	Negative
KI 67 status	Positive (>10%)	Unknown	Unknown	Positive (>10%)
Generation of chemotherapy regimen	Second	Third	Second	Third
				
*5-Year survival results (%)*				
No adjuvant treatment	60	96	76	61
Benefit of adjuvant chemotherapy	15	1	3	18
Benefit of adjuvant Trastuzumab	6	n/a	n/a	n/a
Benefit of adjuvant hormone therapy	n/a	1	4	n/a
Total survival with adjuvant therapy	81	98	83	79
				
*10-Year survival results (%)*				
No adjuvant treatment	49	90	50	49
Benefit of adjuvant chemotherapy	18	1	5	22
Benefit of adjuvant Trastuzumab	5	n/a	n/a	n/a
Benefit of adjuvant Hormone therapy	n/a	2	9	n/a
Total survival with adjuvant therapy	72	93	64	71

Abbreviations: HER2, human epidermal growth factor2.

a Presented with palpable mass; data modified from PREDICT.^5,8^

**Table 3:** Four and 10-year survival using combined Lee–Schonberg calculator in ePrognosis

*Variable*	*Patient 1*	*Patient 2*
Age	75–79 Years	65–69 Years
Gender	Female	Female
BMI	⩾25	⩾25
Patient’s self-reported health	Excellent	Poor
Chronic lung disease	No	No
Prior cancer	No	No
Congestive heart failure	No	Yes
Diabetes or high blood sugar	No	Yes
Describe cigarette use	Never smoked	Current smoker
Difficulty walking a quarter mile without help	No	Yes
Overnight hospitalization in past 12 months	No	Once
Help in routine daily activities	No	No
Memory problems interfering with managing finances	No	No
Memory problem interfering with bathing or showering	No	No
Difficulty pushing or pulling large objects	No	Yes
Estimated 4–5-year survival	96%	77%
Estimated 10-year survival	81%	24%

Abbreviation: BMI, body mass index.

Data modified from www.eprognosis.org.

## References

[bib1] Ravdin, P. M. et al. Computer program to assist in making decisions about adjuvant therapy for women with early breast cancer. J. Clin. Oncol. 19, 980–991 (2001). 11181660 10.1200/JCO.2001.19.4.980

[bib2] Mook, S. et al. Calibration and discriminatory accuracy of prognosis calculation for breast cancer with the online Adjuvant! program: a hospital-based retrospective cohort study. Lancet Oncol. 10, 1070–1076 (2009). 19801202 10.1016/S1470-2045(09)70254-2

[bib3] Campbell, H. E. , Taylor, M. A. , Harris, A. L. & Gray, A. M. An investigation into the performance of the Adjuvant! Online prognostic programme in early breast cancer for a cohort of patients in the United Kingdom. Br. J. Cancer. 101, 1074–1084 (2009). 19724274 10.1038/sj.bjc.6605283 PMC2768087

[bib4] de Glas, N. A. et al. Validity of Adjuvant! Online program in older patients with breast cancer: a population-based study. Lancet Oncol. 15, 722–729 (2014). 24836274 10.1016/S1470-2045(14)70200-1

[bib5] Wishart, G. C. et al. PREDICT: a new UK prognostic model that predicts survival following surgery for invasive breast cancer. Breast Cancer Res. 12, R1 (2010). 20053270 10.1186/bcr2464 PMC2880419

[bib6] Wishart, G. C. et al. A population-based validation of the prognostic model PREDICT for early breast cancer. Eur. J. Surg. Oncol. 37, 411–417 (2011). 21371853 10.1016/j.ejso.2011.02.001

[bib7] Wishart, G. C. et al. PREDICT Plus: development and validation of a prognostic model for early breast cancer that includes HER2. Br. J. Cancer. 107, 800–807 (2012). 22850554 10.1038/bjc.2012.338 PMC3425970

[bib8] PREDICT. PREDICT Tool: Breast Cancer Survival. Available at: http://www.predict.nhs.uk/predict.html (accessed on 22 January 2016).

[bib9] de Glas, N. A. et al. Validity of the online PREDICT tool in older patients with breast cancer: a population-based study. Br J Cancer. 114, 395–400 (2016). 26783995 10.1038/bjc.2015.466 PMC4815772

[bib10] Michaelson, J. S. et al. Improved web-based calculators for predicting breast carcinoma outcomes. Breast Cancer Res. Treat. 128, 827–835 (2011). 21327471 10.1007/s10549-011-1366-9

[bib11] Tang, G. et al. Risk of recurrence and chemotherapy benefit for patients with node-negative, estrogen receptor-positive breast cancer: recurrence score alone and integrated with pathologic and clinical factors. J. Clin. Oncol. 29, 4365–4372 (2011). 22010013 10.1200/JCO.2011.35.3714 PMC3221521

[bib12] Jeruss, J. S. et al. Combined use of clinical and pathologic staging variables to define outcomes for breast cancer patients treated with neoadjuvant therapy. J. Clin. Oncol. 26, 246–252 (2008). 18056680 10.1200/JCO.2007.11.5352

[bib13] Rudloff, U. et al. Nomogram for predicting the risk of local recurrence after breast-conserving surgery for ductal carcinoma *in situ*. J. Clin. Oncol. 28, 3762–3769 (2010). 20625132 10.1200/JCO.2009.26.8847

[bib14] Yourman, L. C. , Lee, S. J. , Schonberg, M. A. , Widera, E. W. & Smith, A. K. Prognostic indices for older adults: a systematic review. JAMA 307, 182–192 (2012). 22235089 10.1001/jama.2011.1966 PMC3792853

[bib15] Jang, R. W. et al. Simple prognostic model for patients with advanced cancer based on performance status. J. Oncol. Pract 10, e335–e341 (2014). 25118208 10.1200/JOP.2014.001457

[bib16] Institute NC. Common Terminology Criteria for Adverse Events (CTCAE) Version 4.0; http://evs.nci.nih.gov/ftp1/CTCAE/CTCAE_4.03_2010-06-14_QuickReference_5x7.pdf.

[bib17] Hurria, A. et al. Predicting chemotherapy toxicity in older adults with cancer: a prospective multicenter study. J Clin Oncol. 29, 3457–3465 (2011). 21810685 10.1200/JCO.2011.34.7625 PMC3624700

[bib18] Extermann, M. et al. Predicting the risk of chemotherapy toxicity in older patients: the Chemotherapy Risk Assessment Scale for High-Age Patients (CRASH) score. Cancer 118, 3377–3386 (2012). 22072065 10.1002/cncr.26646

[bib19] Paik, S. et al. Gene expression and benefit of chemotherapy in women with node-negative, estrogen receptor-positive breast cancer. J. Clin. Oncol. 24, 3726–3734 (2006). 16720680 10.1200/JCO.2005.04.7985

[bib20] Zelnak, A. B. & O'Regan, R. M. Genomic subtypes in choosing adjuvant therapy for breast cancer. Oncology (Williston Park) 27, 204–210 (2013). 23687790

[bib21] Sgroi, D. C. et al. Prediction of late distant recurrence in patients with oestrogen-receptor-positive breast cancer: a prospective comparison of the breast-cancer index (BCI) assay, 21-gene recurrence score, and IHC4 in the TransATAC study population. Lancet Oncol. 14, 1067–1076 (2013). 24035531 10.1016/S1470-2045(13)70387-5 PMC3918681

[bib22] Drukker, C. A. et al. A prospective evaluation of a breast cancer prognosis signature in the observational RASTER study. Int. J. Cancer. 133, 929–936 (2013). 23371464 10.1002/ijc.28082 PMC3734625

[bib23] Wallden, B. et al. Development and verification of the PAM50-based Prosigna breast cancer gene signature assay. BMC Med. Genomics 8, 54 (2015). 26297356 10.1186/s12920-015-0129-6 PMC4546262

